# N6-methyladenosine (m6A)-forming enzyme METTL3 controls UAF1 stability to promote inflammation in a model of colitis by stimulating NLRP3

**DOI:** 10.1038/s41598-025-88435-0

**Published:** 2025-02-18

**Authors:** Yongqiang Lai, Junhao Liu, Xiao Hu, Xiancheng Zeng, Peng Gao

**Affiliations:** 1 The Affiliated Guangdong Second Provincial General Hospital of Jinan University, the Institute of Chest Wall Surgery, Guangzhou, 510700 China; 2 The Affiliated Guangdong Second Provincial General Hospital of Jinan University, the Second Department of General Surgery, Guangzhou, 510317 China

**Keywords:** Methyltransferase-like 3 (METTL3), USP1-associated factor 1 (UAF1), Colitis, m6A, NLR family pyrin domain containing 3 (NLRP3), Cancer, Cell biology, Ecology, Molecular biology

## Abstract

The rising incidence of ulcerative colitis (UC) in China poses a noticeable health challenge. This study aimed to assess the pivotal role of USP1-associated factor 1 (UAF1) in colitis. UC was induced in male C57BL/6 mice using 2.0% dextran sulfate sodium (DSS). In an in vitro model, RAW264.7 cells were exposed to 200 ng/ml of LPS + ATP. UAF1 expression level was evaluated in colonic tissues, macrophages, and serum samples using quantitative reverse transcription polymerase chain reaction (RT-qPCR). The study assessed weight, disease activity index (DAI) score, myeloperoxidase (MPO) activity, crypt length, inflammatory factors, and epithelial cell function in a mouse model of colitis treated with a UAF1 inhibitor. Microarray analysis identified potential UAF1 targets. Gene interference investigated NLR family pyrin domain containing 3 (NLRP3) involvement in UAF1-induced colitis inflammation. Immunoprecipitation, ubiquitination, and luciferase assays examined the effects of methyltransferase-like 3 (METTL3) methylation on the expression levels of NLRP3 and UAF1. UAF1 expression level was upregulated in colon tissues, RAW264.7 macrophages, and serum samples of colitis mice (*P* < 0.01). The UAF1 inhibitor (ML-323) enhanced weight and reduced DAI score in colitis mice (*P* < 0.01). It also decreased MPO activity and ulcer area, and restored crypt length (*P* < 0.01). UAF1 inhibitor improved epithelial cell function by suppressing NLRP3 activity (*P* < 0.01). UAF1 promoted inflammation in RAW264.7 macrophages via NLRP3 inflammasome induction (*P* < 0.01). UAF1 modulated NLRP3 protein expression, leading to reduced NLRP3 ubiquitination induced by LPS + ATP. The m6A-forming enzyme METTL3 enhanced UAF1 stability (*P* < 0.01) to facilitate UAF1 expression. The findings suggested that METTL3, as an m6A-forming enzyme, could regulate UAF1 mRNA, promoting inflammation in colitis through NLRP3 induction. Inhibiting UAF1 emerges as a potential therapeutic strategy for colitis.

**Supplementary Information:**

The online version contains supplementary material available at 10.1038/s41598-025-88435-0.

## Introduction

Inflammatory bowel disease (IBD) is characterized by the development of intestinal mucosal ulcers and the progressive deterioration of intestinal function^1^. The impact of IBD extends beyond health, imposing a substantial financial and resource burden on the healthcare system^2–4^. Emerging evidence underscores the role of intestinal flora imbalance in disrupting intestinal bile acid metabolism as a contributing factor to IBD^5^.

Chronic colitis lacks a standardized nomenclature and primarily presents with symptoms, such as diarrhea, abdominal pain, and occasionally the presence of mucous pus and blood in stools^6^. Despite colonoscopic observations revealing chronic inflammatory changes, the absence of distinctive features precludes a narrow diagnosis of ulcerative colitis (UC)^6^. The lesions tend to stabilize at a certain stage without significant progression, with several cases exhibiting recurring patterns that may persist for a lifetime without substantial recovery^7^. Current Western medicine offers limited pharmaceutical options for chronic colitis^7^. Mild cases mainly involve seeking the cause and symptomatic treatment^8^. The conservative measures are the primary approach for chronic cases. In cases of explosive or inadequately managed internal medicine, surgical interventions are employed, while their clinical efficacy is often unsatisfactory^9^. Moreover, there is a lack of prognostic insight into the progression of this disease^9^. If left unaddressed, recurrent episodes pose risks of exacerbation, potential cancer transformation, development of UC, formation of benign protrusions (e.g., inflammatory polyps), or a gradual transformation into colon cancer^10^.

UC predominantly influences the rectum and colon, presenting with recurrent symptoms characterized by intermittent diarrhea, mucopurulent bloody stools, abdominal pain, and urgency^11^. The specific cause of UC remains uncertain^11^, and its etiology that could be influenced by various factors, including environmental factors, genetics, intestinal microbiota, and immunity^12^. The condition has a protracted course, marked by recurring gastrointestinal disturbances, significantly impacting daily life. Patients mainly experience physiological and psychological challenges^13,14^, emphasizing the positive role of psychological health in ameliorating UC^15^.

Global statistics indicated that the prevalence of UC in developed countries exceeds 0.3%, with a rapidly rising incidence rate in China and other recently industrialized nations^16^. Predictions suggest that by 2025, UC will influence up to 30 million people worldwide. Clinical manifestations include abdominal pain, diarrhea, acute diarrhea, mucous pus, bloody stools, and, in some cases, systemic symptoms^17^.

In colitis, the NLR family pyrin domain containing 3 (NLRP3) inflammasome is activated and assembled, contributing to maturation processes^18–20^. Pyroptosis, a key element in the classical pyroptosis pathway mediated by Caspase-1, involves cell activation by external stimuli, triggering the intracellular sensor NLRP3^21,22^. The inflammasome binds to Caspase-1 precursor, leading to its cleavage and activation. Activated Caspase-1 exhibits pore-forming activity, specifically facilitating the release of intracellular inflammatory factors, thereby resulting in a robust inflammatory response^18^.

Maintaining the normal physiological activities of cells, including the regulation of cell cycle arrest, DNA repair, aging, and apoptosis, relies on the intricate process of DNA damage repair^23^. Abnormalities in this crucial mechanism may lead to various pathological outcomes^23^. Research indicated that modulating the level of N6-methyladenosine (m6A) in cells can induce differential expression of genes associated with DNA damage repair^24^. M6A, the most frequent and abundant RNA modification in eukaryotic mRNA, exerts its influence on cellular behavior through diverse biological mechanisms^25^. When its level is aberrant, it can contribute to tumor proliferation, metastasis, differentiation, and immune escape. Methyltransferase-like 3 (METTL3), a key enzyme involved in m6A modification, plays a pivotal role in various diseases^26^.

USP1-associated factor 1 (UAF1) regulates the enzymatic activity of the USP subfamily complex, leading to the deubiquitination of a series of substrate proteins and influencing tumor development and antiviral immune responses^27–29^. In murine embryonic fibroblasts, UAF1 and NLRP3 could be colocalized after LPS stimulation^30,31^. However, the pathogenesis of UAF1 remains incompletely understood, and current treatment strategies are mainly ineffective. The methylation status of certain genes is correlated with atypical hyperplasia of UC and even UC-related colon cancer. Some genes exhibit significant differences in methylation levels between UC and other diseases, with abnormal methylation occurring exclusively in UC. Therefore, UAF1 may regulate inflammation in UC through its interaction with NLRP3. The present study aimed to assess the critical role of UAF1 in colitis, its potential upstream key regulator METTL3, and the underlying mechanisms driving inflammation. In the landscape of IBD research, there exists a pivotal insufficiently examined domain concerning the molecular complexities that regulate chronic colitis. Despite considerable advancements in understanding IBD, a gap is noteworthy in the comprehension of the specific molecular mechanisms underlying the persistent nature of chronic colitis. This study addressed this gap by focusing on the role of UAF1 and its potential interaction with NLRP3 in the context of colitis. The lack of clarity on how UAF1, along with its upstream regulator METTL3, contributes to the inflammatory processes in chronic colitis underscores the need for a comprehensive investigation. Bridging this gap is imperative for advancing our understanding of colitis progression and, consequently, for the development of targeted therapeutic interventions.

## Materials and methods

### Mouse model

Male C57BL/6 mice (**4–5 weeks**,** 17–19 g**) were purchased from Animal Experimental Center of Guangdong Medical University (Guangzhou, China). Colitis was induced by administering 2.0% dextran sulfate sodium (**DSS**,** D8906; Sigma-Aldrich**,** St. Louise**,** MO**,** USA**) in the drinking water for 7 days. Mice were provided with a standard diet and water ad libitum. The present study was approved by the Animal Care and Use Committee of Guangdong Second Provincial General hospital (Approval No. 2021-KZ-235-04). UAF1 inhibitor (10 mg/kg/day for 7 days of ML-323, HY-17543; MedChemExpress) was injected. Anesthesia was induced with 50 mg/kg pentobarbital sodium, and mice were euthanized by cervical dislocation. Colon tissues, RAW264.7 macrophages, and serum samples were extracted as described previously^32,33^.

### Histological analysis

Colon tissue samples were fixed with 4% paraformaldehyde and sectioned into 5 μm slices for histological analysis, immunohistochemistry (IHC), and immunofluorescence (IF), as described in previous studies^3,33^. Hematoxylin and eosin (HE) staining or Alcian blue staining were performed on colon tissues. For HE staining, tissue sections were immersed in a buffered solution with a defined pH before staining and stained with hematoxylin, followed by controlled staining time under a microscope, ensuring clearly stained nuclei and essentially colorless cytoplasm. The tissues were stained with alcohol eosin staining solution for 2–3 min. Alternatively, tissue sections were stained and soaked in Alcian acidifying solution and Alcian staining solution sequentially, rinsed with running water, and subsequently counterstained with nuclear fast red staining solution, followed by rinsing with running water. Tissue sections were gradually dehydrated through a series of alcohol concentrations and cleared with xylene. The transparent sections were mounted onto neutral resin and sealed with a cover slip.

### IHC and IF

The sulfomucin ratio and the count of sulfomucin-positive goblet cells were determined according to the previously described method^34^. Tissue sections were subjected to 5-min incubation in 100% methanol (previously frozen at – 20 °C) at room temperature. Thereafter, the samples were treated with PBS, containing 0.1 to 0.25% Triton X-100 for 10 min. Subsequently, cells underwent thrice washing with PBS each lasted for 5-min. To block nonspecific antibody binding, cells were treated with a solution of 1% BSA and 22.52 mg/mL glycine in PBST (PBS + 0.1% Tween 20) for 30 min. Colon tissues were subsequently incubated with UAF1 antibody (ab230645, 1:100; Abcam, Cambridge, UK) or Muc-2 antibody (ab272692, 1:100; Abcam) at 4 °C overnight. After washing with PBS, cells were incubated with fluorescent conjugated secondary antibody, dissolved in 1% BSA, in the dark for 1 h at room temperature. Following this, samples were incubated with DAPI (DNA staining) for 1 min. Observation of colon tissues was carried out using a fluorescence microscope (IX71; Olympus, Tokyo, Japan) after washing with PBS. For each sample, five different random views were selected for photography, and ImageJ software was utilized to calculate the fluorescence intensity of various colors.

### Immunohistochemistry (IHC)

Tissue sections were immersed in 100% methanol (previously frozen at – 20 °C) for 5 min at room temperature. Subsequently, the samples underwent 10-min incubation with PBS, containing 0.25% Triton X-100. Colon tissues were then stained with UAF1 (ab230645, 1:100, Abcam) at 4 °C overnight. Following washing with PBS, the tissue sections were incubated with a horseradish peroxidase (HRP)-conjugated secondary antibody labeled with enzyme substrates to generate staining signals. Finally, colon tissues were observed under a normal light microscope (IX71, Olympus) after being washed with PBS.

### Immunofluorescence staining (IF)

Tissue sections were immersed in 100% methanol (previously frozen at – 20 °C) for 5 min at room temperature. Following this, the samples underwent 10-min incubation with PBS, containing 0.25% Triton X-100. Colon tissues were then incubated with Muc-2 antibody (ab272692, 1:100, Abcam) at 4 °C overnight. After washing with PBS, the tissues were incubated with Alexa Fluor 488-labeled Goat anti-rabbit IgG (H + L) antibody. Subsequently, samples were incubated with DAPI (DNA staining) for 1 min. Colon tissues were observed under a fluorescence microscope (IX71, Olympus) after being washed with PBS.

Cells were incubated with 4% paraformaldehyde for 20 min, followed by incubation with PBS, containing 0.25% Triton X-100 for 10 min. Colon tissues were incubated with UAF1 and Muc-2 antibodies (ab272692, 1:100, Abcam) at 4 °C overnight. After washing with PBS, cells were incubated with fluorescent or HRP-conjugated secondary antibody labeled with enzyme substrates to generate staining signals. Colon tissues were observed under a fluorescence microscope (IX71, Olympus) after being washed with PBS.

### Microarray analysis

Double-stranded cDNA synthesis was carried out utilizing a NimbleGen one-color DNA labeling kit, followed by array hybridization using the NimbleGen hybridization system and subsequent washing with the NimbleGen buffer. The scanning process was performed with the Axon GenePix 4000B microarray scanner (Molecular Devices, New York, NY, USA). Data collection was conducted using Feature Extraction 10.7 software (Agilent Technologies). Heatmap/volcano plotting and gene set enrichment analysis (GSEA) were undertaken employing Gene Spring 11.0 software (Agilent Technologies). Raw data underwent normalization through the Quantile algorithm in the limma R package. Microarray analysis and drawing volcano maps and heatmaps were performed by Shanghai Biotechnology Corp. (Shanghai, China). Genes with incomplete annotation information, very low expression levels, and duplications were systematically filtered out. Differentially expressed mRNAs were selected based on screening conditions of false discover rate (FDR) < 0.05 and |log fold-change| > 2.5. Target gene prediction utilized the miWalk web tool, and the signaling pathways associated with the identified genes underwent enrichment analysis. GSEA and Gene Ontology (GO) enrichment analysis were employed to analyze upregulated and downregulated genes, respectively, using the clusterProfiler R package.

### In vitro model

RAW264.7 cells (SCSP-5036; Cell Bank of the Chinese Academy of Sciences), which confirmed to be negative for mycoplasma, were maintained at 37 °C under 5% CO_2_ in a Dulbecco’s modified Eagle’s medium (DMEM) supplemented with 10% fetal calf serum (FCS, 26170035; Invitrogen, Carlsbad, CA, USA). Lentiviral constructs for UAF1 and METTL3 knockdown or overexpression were obtained from OBIO (Obio Technology Corp., Shanghai, China). Lentiviral vectors carrying siRNA for UAF1 and METTL3 knockdown, as well as overexpression, along with a negative control, were synthesized and cloned into the pLKO.1 vector. Cells were transfected with these plasmids using Lipofectamine 3000 reagent (L3000008, Invitrogen). The sequences of siRNAs used in the study were summarized as follows:

-si-UAF1: Forward, CTAGTCTAGAATGGCGGCCCATCAC; Reverse, CCGCTCGAGCGTGGACTTCTGACGGTAA.
-si-METTL3: Forward, 5′-AAGCTGCACTTCAGACGAAT-3′; Reverse, 5′-GGAATCACCTCCGACACTC-3′.
-si-nc (negative control): Forward, 5′-GGUCGAGACUCCAUCAUAA-3′; Reverse, 5′-CCCCCCCCCCCCCC-3′.

After 48 h, RAW264.7 cells were treated with 200 ng/ml LPS (L5293, Sigma-Aldrich) for 4 h plus 1 mM ATP (FLAAS, Sigma-Aldrich) for 30 min.

### Luciferase assay

The luciferase activity was measured using the Dual-Glo Luciferase reporter assay kit (E2920; Promega, Madison, WI, USA) according to the manufacturer’s protocol. Briefly, the Dual-Glo® Luciferase assay reagents were added to the plate, and incubated at 20–25 °C for 1 h. The firefly luminescence was measured. Subsequently, the Dual-Glo® Stop & Glo reagent was added to the plate, and the Renilla luminescence was measured. In the luciferase activity assessment of wild-type and mutant UAF1, the first step involved cloning the UAF1 promoter region into a luciferase reporter plasmid and generating wild-type and mutant UAF1 expression plasmids. Following this, cells were cultured and transfected with the luciferase reporter plasmid along with wild-type and mutant UAF1 plasmids. After 48-h incubation, cells are lysed, and luciferase activity was measured. The obtained data were analyzed to determine significant differences in luciferase activity between wild-type UAF1 and mutant UAF1.

### Quantitative reverse transcription polymerase chain reaction (RT-qPCR)

RT-qPCR was conducted using the ABI Prism 7500 sequence detection system following the Prime-ScriptTM RT detection kit’s protocol (RR037; Takara, Shiga, Japan). The relative levels of mRNA expression in the samples were calculated using 2^−ΔΔCT^ method. β-actin gene was utilized as an internal reference. The following primers were used (5′ to 3′):

UAF1: forward, cgttcaacttgcctgtgctaatcaac;

reverse, cgcaaattttacgatt ttggagtcaaa;

Cxcl1: forward, CACCCAAGTCATAGCAAACCG;

reverse, GAAGCGCGTTCACCAGACA;

Cxcl2: forward, GACAGAAGTCAGCCACTCTCAT;

reverse, GCCTTGCCTTCAGTATCTTGT;

TFF3: forward, TTGCTGGATAGGGGTCCTCTG;

reverse, TACACGATGTGACAGTGCTCC;

RELMβ: forward, CCCTTTCA ACCTCCAGCTGA;

reverse, CCACGATAGAACCACAGCC;

Saa1: forward, ACACTAACCTGACATGAAGGAAG;

reverse, CATTCCTGACTCTGCCGAAGA;

Saa3: forward, AGGTTCCAAAGATGGGTCCA;

reverse, TCATAGAGTGCCACAGGCTC;

Reg3b: forward, AATATGGGAATGGGAGGTGG;

reverse, CCACGTCTAAAGAAAGCACG;

Reg3g: forward, CTTCATGATCAAACTGTCCTCC;

reverse, CCACCTGTTCATAGCTGTTGG;

Defb1: forward, TCATCTGTCAGCCCAACTACC;

reverse, CGGAGACAGAATCCGTTCCAT;

Defa2: forward, GGCAATTCTTCCTGCTCACC;

reverse, GATCACCTGGAAGGCCTGGA;

Defa3: forward, TCGGAGACCACCTGAACATG;

reverse, CGACAGGCACCGGTAGTCAT;

Defa21: forward, CGAGATGACACTGAGAGTGC;

reverse, GAAGGCCCCAAGTGTTCATC;

Defa24: forward, ACACACTCACCTGAGCTGCT;

reverse, AGACACCTCTTCAGCCTGGT.

GAPDH: forward, TCAAGG CTGAGAACGGGAAG;

reverse, TCGCCCCACTTGATTTTGGA.

### Enzyme-linked immunosorbent assay (ELISA)

IFN-γ (H025-1-2), TNF-α (H052-1-2), MPO (A044-1-1), IL-17 (H014-1), IL-6 (H007-1-1), and IL-1β (H002-1-2) kits obtained from Nanjing Jiancheng Bioengineering Research Institute (Nanjing, China) were utilized to quantify the levels of cytokines in accordance with the manufacturer’s instructions.

### Western blotting

Western blotting was performed as previously described^21^. Membranes were incubated with UAF1 (ab230645, 1:1000; Abcam), NLRP3 (sc-134306, 1:1000; Santa Cruz Biotechnology, Santa Cruz, CA, USA), and β-Actin (BS6007MH, 1:5000; Bioworld Technology, Inc., St Louis Park, MN, USA) at 4 °C overnight. The membranes were incubated with HRP-conjugated secondary antibodies (sc-2004 or sc-2005, 1:5000; Santa Cruz Biotechnology) for 1 h at 37 °C after washing with TBST for 15 min. Protein was measured using a ChemiDoc Touch system (Bio-Rad Laboratories, Inc., Hercules, CA, USA) and analyzed using Image Lab 3.0 software (Bio-Rad Laboratories, Inc.).

### Immunoprecipitation assay (IP)

The ***IP*** experiment was conducted as previously described^35^. Cells were lysed with RIPA lysis buffer (HY-K1001, MedChemExpress), and the supernatant was incubated with antibody- or rabbit IgG-conjugated protein A/G magnetic beads (HY-K0202, MedChemExpress) in IP buffer supplemented with RNase inhibitors (HY-K1033, MedChemExpress) overnight at 4 °C. Protein A was added to capture the antibody-target protein-DNA complex. The precipitated complexes were washed to eliminate nonspecific binding. The enriched target protein-DNA complexes were obtained through elution. Following decrosslinking, the enriched DNA fragments were purified, and the isolated DNA fragments were subjected to qPCR analysis.

### mRNA stability assay

After transfected for 24 h, RAW264.7 cells were treated with 2 µg/mL actinomycin D (Sigma–Aldrich, Louis, MO) to inhibit global mRNA transcription. After incubation at the hour 0, 3, 6 and 9, cells were collected, and RNA was isolated for qPCR detection as described above to assess degradation.

### Statistical analysis

Student’s t-test or one-way analysis of variance (ANOVA) was used for statistical analysis of the data expressed as mean ± standard error of the mean (SEM). *P* < 0.05 was considered statistically significant.

## Results

### UAF1 expression was up regulated in epithelial cells of colitis mice

**Fig. 1:**
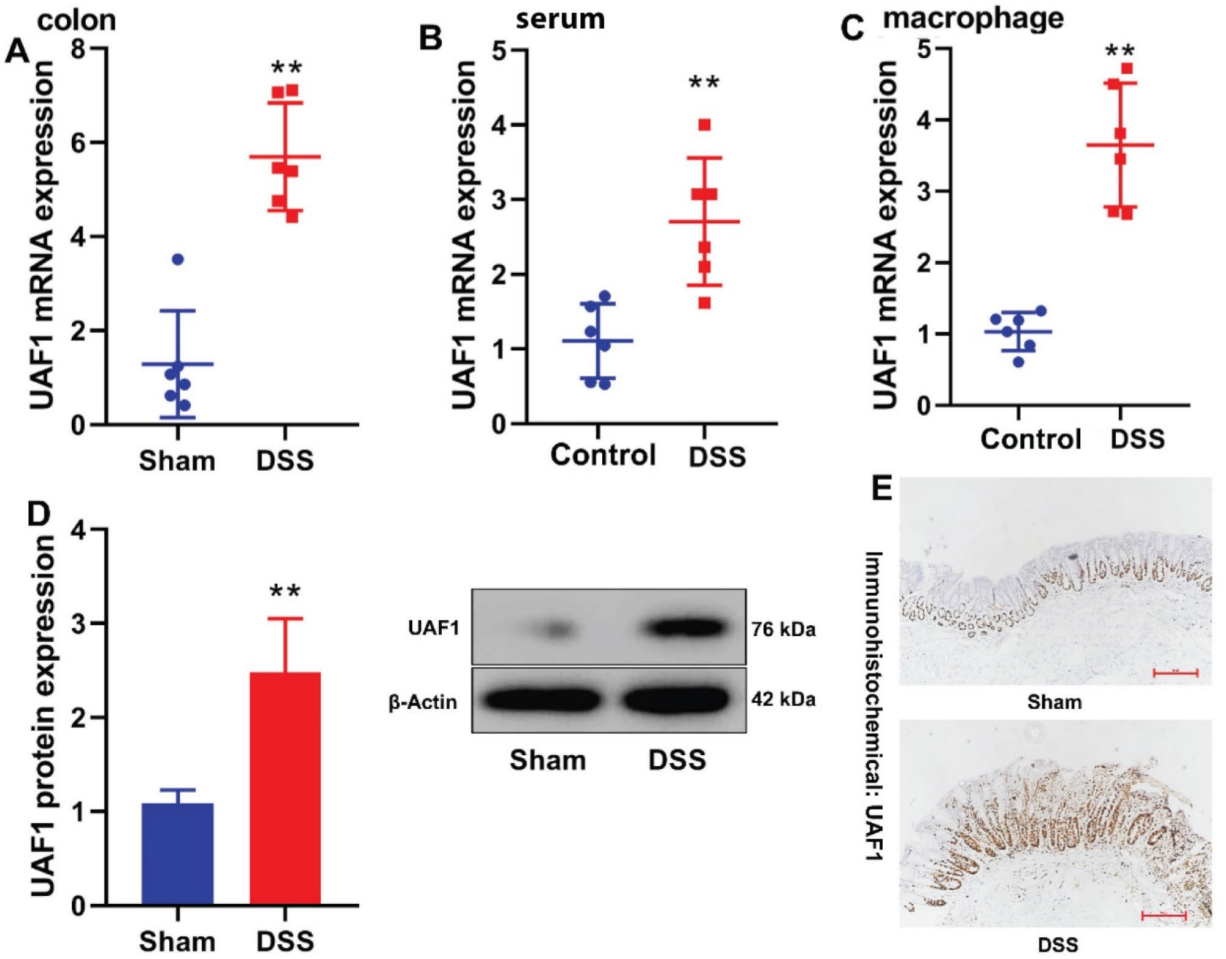
UAF1 expression in mice with UC. UAF1 mRNA expression in colon tissues, serum samples, and RAW264.7 macrophages (A, B, C) of mice with colitis; UAF1 protein expression (D) in colon tissues of mice with colitis; UAF1 protein expression (IHC, E, scale bar = 500 μm) in colon tissues of mice with colitis. Sham, sham control group; DSS, DSS-induced colitis group. **P < 0.01 compared with Sham or Control group (*n* = 6).

The present study assessed the UAF1 expression level in mice with colitis through RT-qPCR. UAF1 mRNA expression level in colon tissues, RAW264.7 macrophages, and serum samples of mice with colitis was significantly upregulated (*P* < 0.01, Fig. 1A–C). Additionally, UAF1 protein expression level in colon tissue was significantly elevated in the mouse model of colitis (*P* < 0.01, Fig. 1D). IHC further revealed an upregulation of UAF1 expression level in colon tissue in the mouse model of colitis (Fig. 1E).

### UAF1 inhibitor reduced the levels of inflammatory markers in the mouse model of colitis

**Fig. 2:**
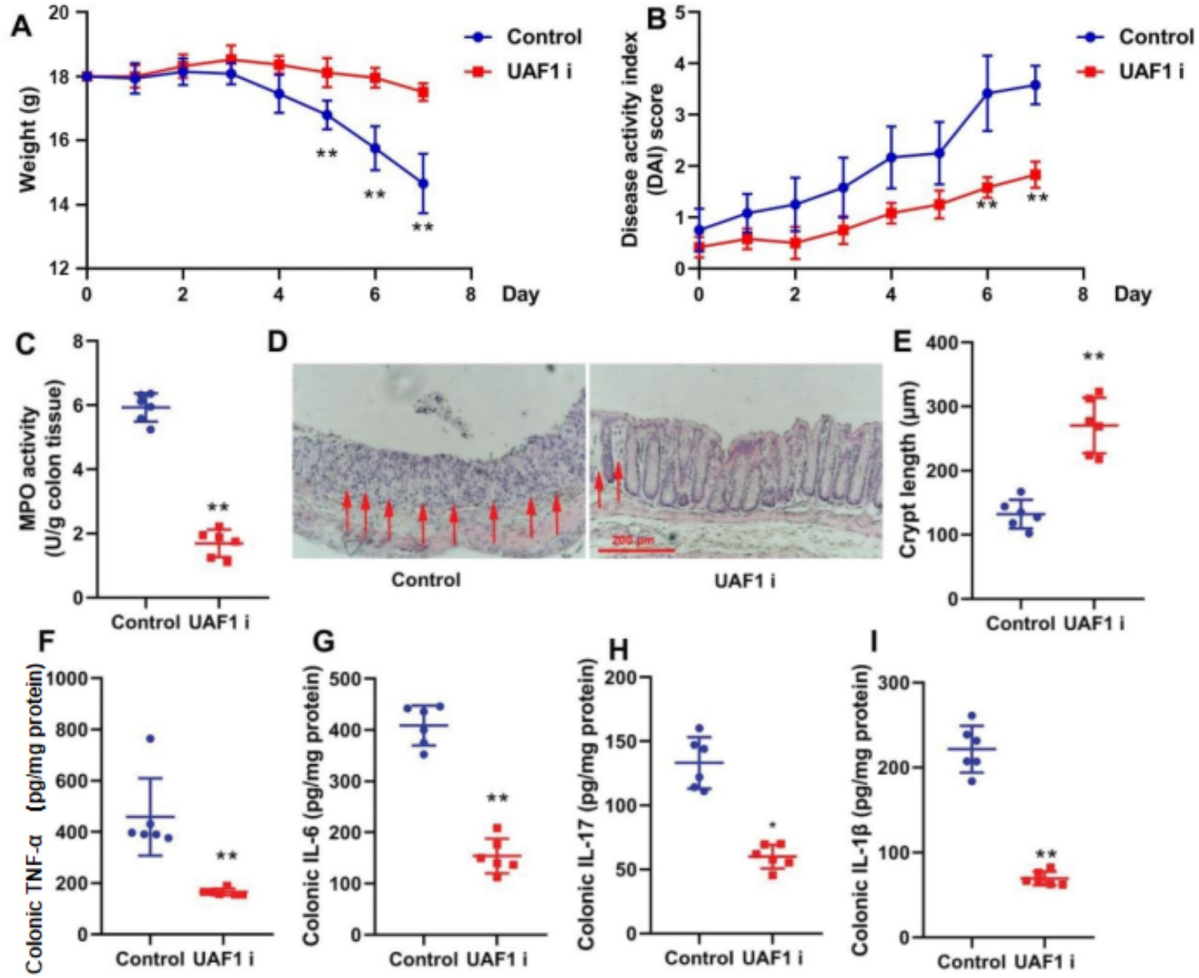
The inhibition of UAF1 reduced inflammation in the mouse model of colitis. Weight (A), DAI score (B), MPO activity level (C), ulcer area (HE staining, D, the points of MPO inhibition in the ulcer area were marked with arrows), crypt length (E), and TNF-α, IL-6, IL-17, and IL-1β levels (F, G, H, and I) in mice with colitis. Control group: normal; colitis group: induced colitis; UAF1i group: colitis treated with UAF1 inhibitor; *P < 0.05 compared with Control group; **P < 0.01 compared with Control group (*n* = 6).

It was attempted to examine whether UAF1 could regulate the levels of inflammatory markers in the mouse model of colitis. Treatment with the UAF1 inhibitor (ML-323, 10 mg/kg) significantly increased body weight and reduced DAI score in the mouse model of colitis (*P* < 0.01, Fig. 2A, B). The UAF1 inhibitor significantly inhibited MPO activity in colon tissue and ulcer area, while significantly recovered crypt length in the mouse model of colitis (*P* < 0.01, Fig. 2C–E). Additionally, the UAF1 inhibitor demonstrated a significant suppression of the levels of inflammatory markers (TNF-α, IL-6, IL-17, and IL-1β) in the colon tissue of colitis mice (*P* < 0.05 and *P* < 0.01, Fig. 2F–I).

### The inhibition of UAF1 recovered epithelial cell function in the mouse model of colitis

**Fig. 3:**
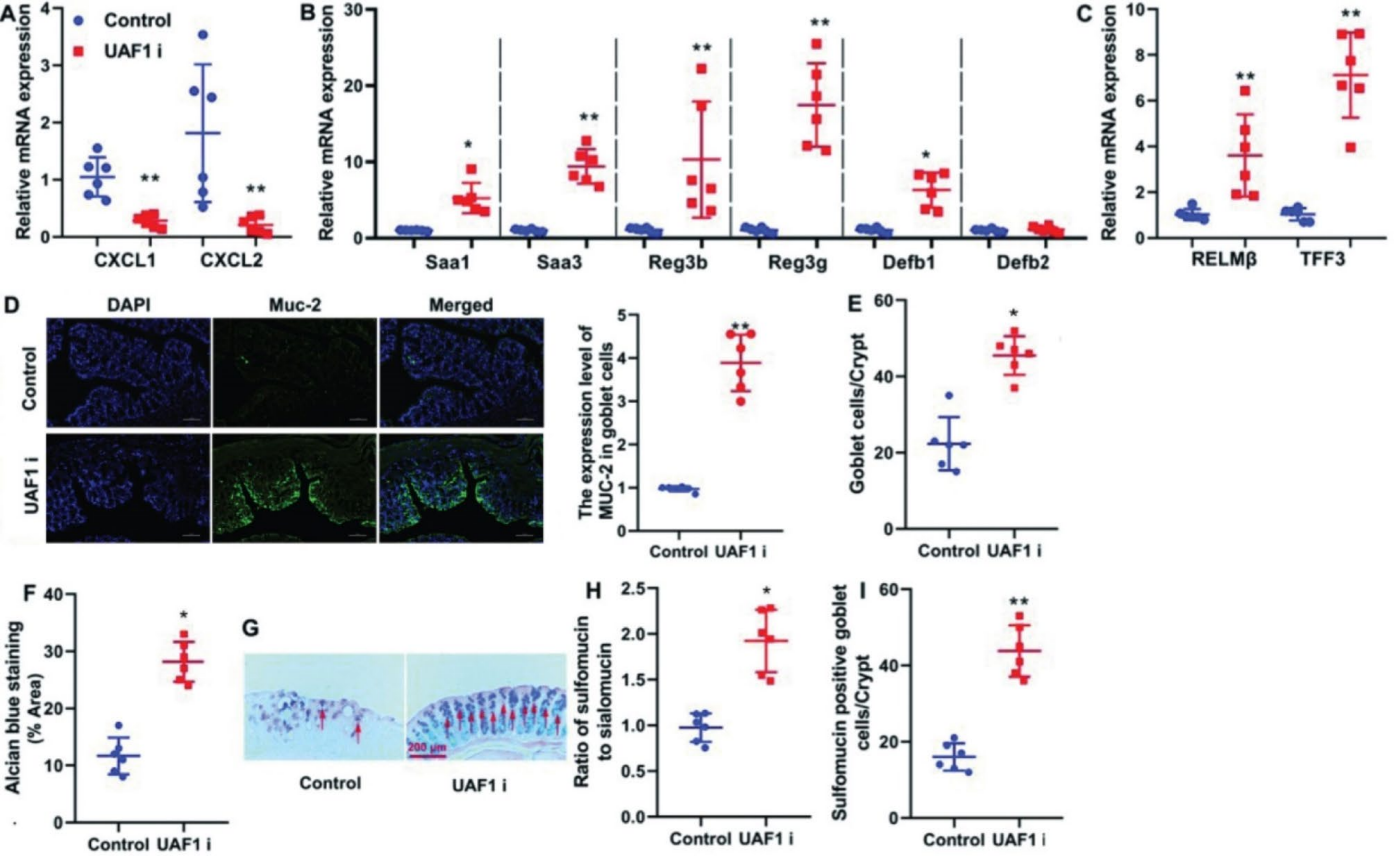
The inhibition of UAF1 recovered epithelial cell function in the mouse model of colitis. CXCL1/CXCL2 mRNA expression level (A), Saa1, Saa3, Reg3b, Reg3g, Defb1, and Defb2 mRNA expression levels (B), RELMβ and TFF3 mRNA expression levels (C), MUC-2 level (D, scale bar = 200 μm), ratio of goblet cells to crypt (E), Alcian blue staining (F), Alcian blue/HE staining (G), ratio of sulfomucin/sialomucin (H, Alcian blue staining), and sulfomucin positive goblet cells/Crypt (I). Control, DSS-induced colitis group; UAF1i, DSS-induced colitis by UAF1 inhibitor group; *P < 0.05 compared with Control group; **P < 0.01 compared with Control group (*n* = 6).

Subsequently, the study concentrated on determining whether the UAF1 inhibitor could effectively restore epithelial cell function in the mouse model of colitis. The UAF1 inhibitor demonstrated a significant reduction in CXCL1 and CXCL2 mRNA expression levels, as well as a remarkable increase in Saa1, Saa3, Reg3b, Reg3g, Defb1, and Defb2 mRNA expression levels, which are crucial homeostatic molecules in the colon epithelium. Furthermore, it notably enhanced RELMβ and TFF3 mRNA expression levels, which are key factors associated with goblet cell secretion, in the colon tissue of colitis mice (*P* < 0.01, Fig. 3A–C). The UAF1 inhibitor effectively restored alcian blue staining and goblet cells, while elevated Muc-2 level in the colon tissue of colitis mice (*P* < 0.05 and *P* < 0.01, Fig. 3D–G). Additionally, the UAF1 inhibitor significantly increased the number of sulfomucin-positive goblet cells in the colon tissue of colitis mice (*P* < 0.05, Fig. 3H). **Notably**,** determining the ratio of sulfomucin to sialomucin involved initially collecting mucin samples**,** followed by the isolation of mucins through techniques**,** such as centrifugation or chromatography. Quantification of sulfomucins and sialomucins was achieved**,** and the ratio was subsequently calculated by dividing the amount of sulfomucins by the amount of sialomucins.** Hence, the inhibition of UAF1 emerged as a robust strategy for the substantial restoration of epithelial cell function in the mouse model of colitis through effectively inflammation suppression.

### The inhibition of UAF1 suppressed NLRP3-induced IL-1β release in the mouse model of colitis

**Fig. 4:**
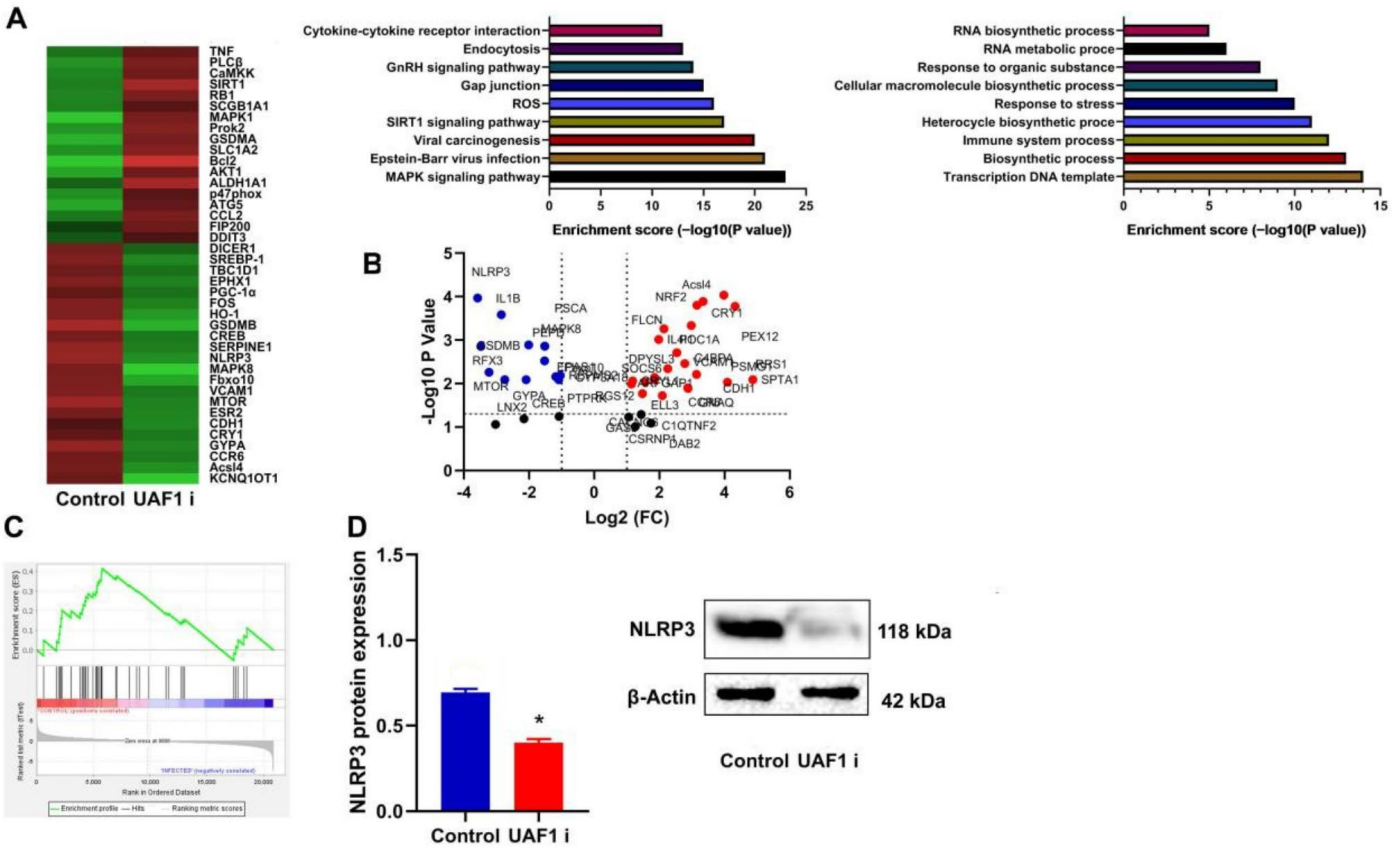
NLRP3 and IL-1β are potential targets of UAF1 in the mouse model of colitis. The results of microarray analysis are visualized through a heat map depicting differentially expressed genes and enriched pathways (A), a volcano map highlighting gene expression changes (B), GSEA enrichment graph illustrating the NLRP3 inflammasome signaling pathway (C), and NLRP3 protein expression in the colon tissue of mice with colitis (D). The experimental groups include the Control (DSS-induced colitis group) and UAF1i (DSS-induced colitis treated with UAF1 inhibitor group); **P < 0.01 compared with Control group (*n* = 3).

It was further attempted to examine the mechanism of UAF1 on inflammation in the mouse model of colitis using microarray analysis. The heat map (Fig. 4A), volcano map (Fig. 4B), and GSEA enrichment graph (Fig. 4C) suggested that NLRP3, as a differentially expressed gene, could play a crucial role in UFA1-promoted inflammation progression in the mouse model of colitis. NLRP3 and IL-1β genes were mapped in this intersection. Thus, NLRP3 protein expression level was determined and it was found that UAF1 inhibitor markedly restrained NLRP3 protein expression level in colon tissue of colitis mice (*P* < 0.05, Fig. 4C and D).

### UAF1 promoted inflammation in RAW264.7 macrophages through induction of NLRP3 inflammasome

**Fig. 5:**
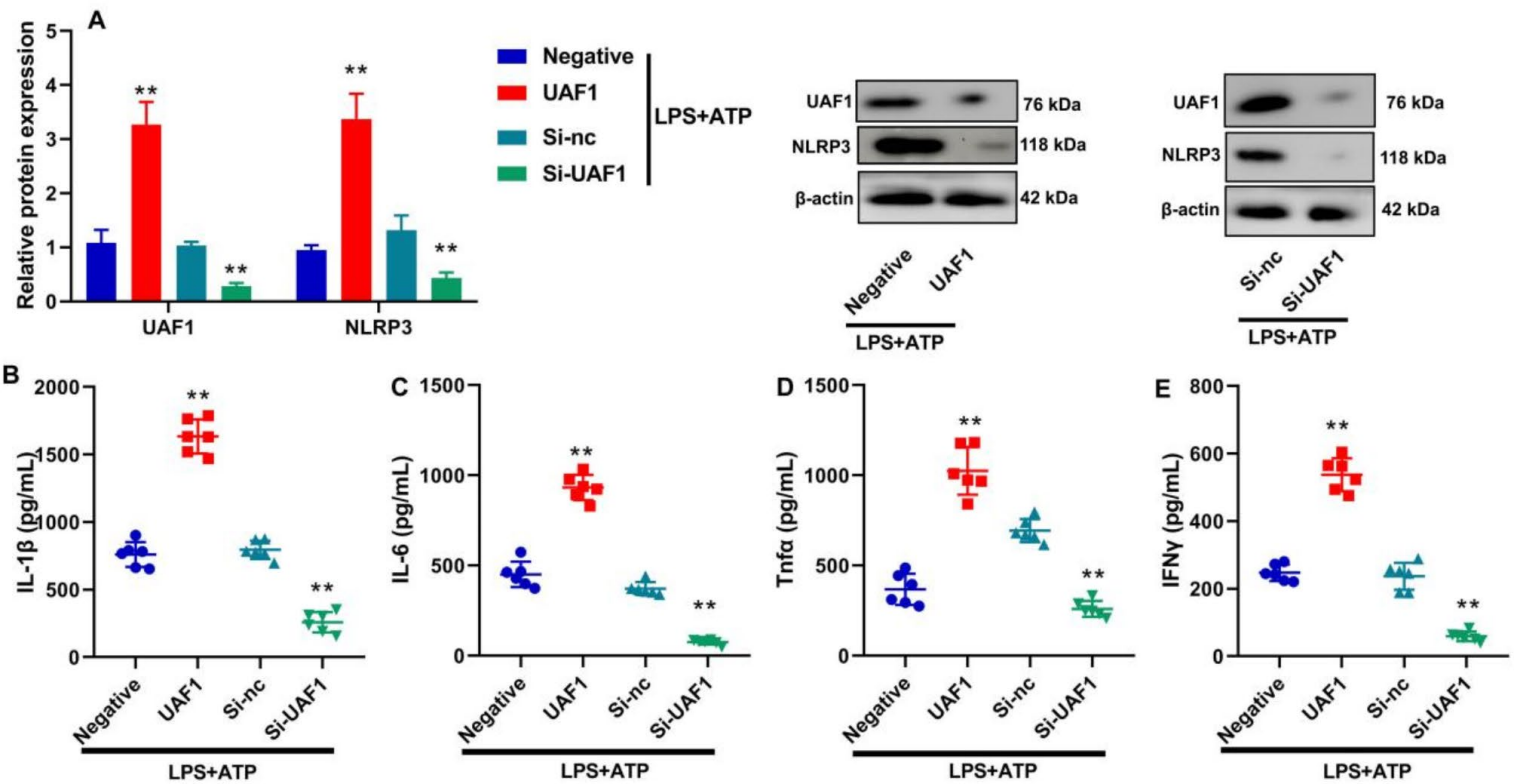
UAF1 promoted inflammation in RAW264.7 macrophages by LPS + ATP through induction of NLRP3 inflammasome. UAF1 and NLRP3 protein expression levels (A); IL-1β, IL-6, TNF-α, and IFN-γ levels (B, C, D, and E). **P < 0.01 compared with negative or Si-nc group (*n* = 6).

Subsequently, RAW264.7 macrophages were subjected to LPS + ATP treatment. Overexpression of UAF1 significantly elevated NLRP3 protein expression level, whereas the knockdown of UAF1 using siRNA markedly reduced NLRP3 protein expression level (*P* < 0.01, Fig. 5A). UAF1 played a pivotal role in elevating the levels of inflammatory markers in RAW264.7 macrophages stimulated by LPS + ATP (*P* < 0.01, Fig. 5B and E).

**Fig. 6:**
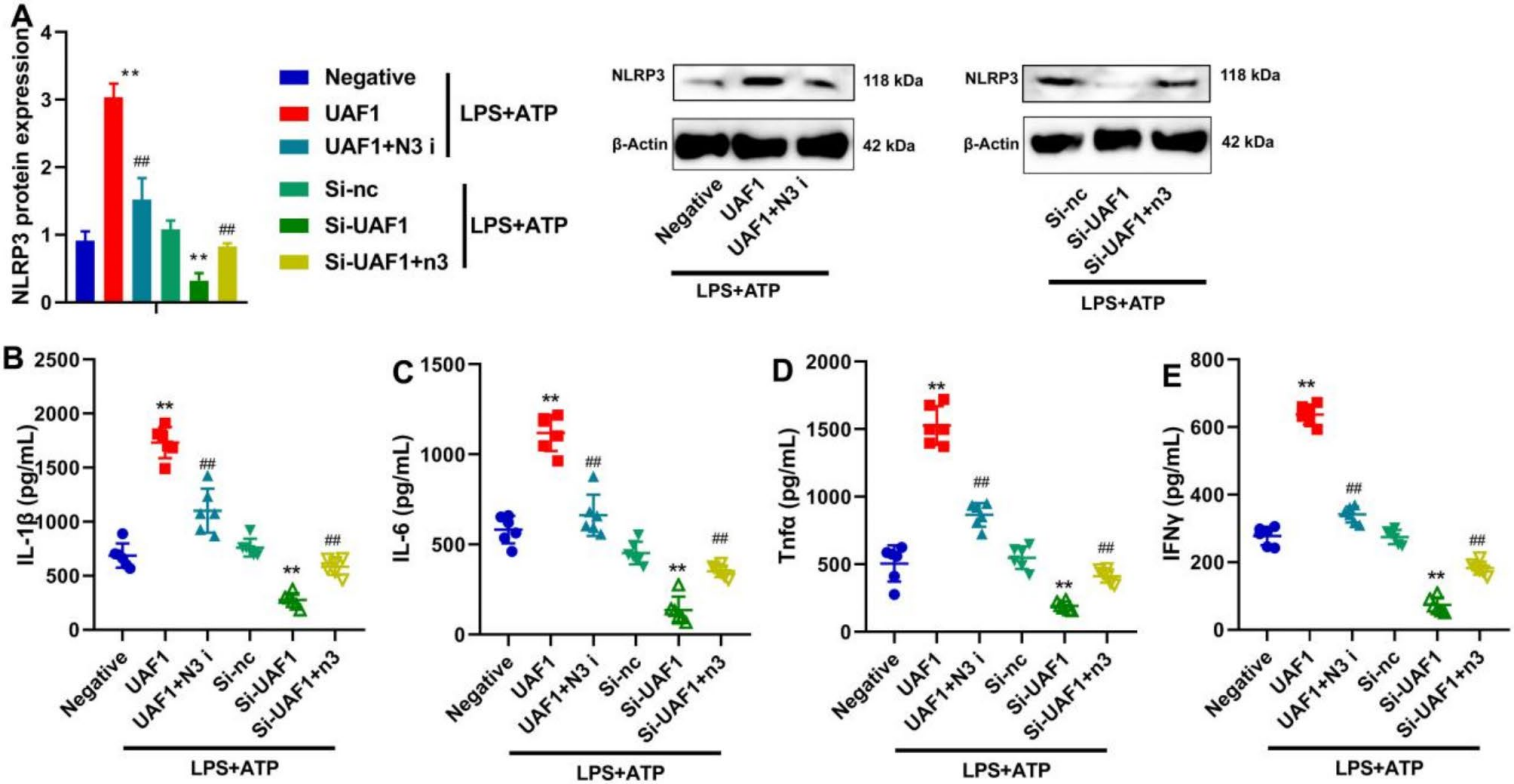
The regulation of NLRP3 could control the effects of UAF1 on inflammation in RAW264.7 macrophages by LPS + ATP. UAF1 and NLRP3 protein expression levels (A); IL-1β, IL-6, TNF-α, and IFN-γ levels (B, C, D and E). N3, NLRP3 agonist; N3 i, NLRP3 inhibitor; **P < 0.01 compared with negative or Si-nc group; ^##^*P* < 0.01 compared with UAF1 or Si-UAF1 group (*n* = 6).

It was revealed that NLRP3 inhibitor (2 µM of CY-09) markedly suppressed NLRP3 protein expression level, and significantly reduced the levels of inflammatory markers in LPS + ATP-induced RAW264.7 macrophages by UAF1 overexpression (*P* < 0.01, Fig. 6A). NLRP3 agonist (5 µM of Nigericin) significantly induced NLRP3 protein expression level, and significantly increased the levels of inflammatory markers in LPS + ATP-induced RAW264.7 macrophages by UAF1 downregulation (*P* < 0.01, Fig. 6A). Collectively, the findings demonstrated that UAF1 promoted inflammation in RAW264.7 macrophages by LPS + ATP through the induction of NLRP3 inflammasome (*P* < 0.01, Fig. 6B-E).

### UAF1 interlinking NLRP3 protein expression to reduce NLRP3 ubiquitination by LPS + ATP

**Fig. 7:**
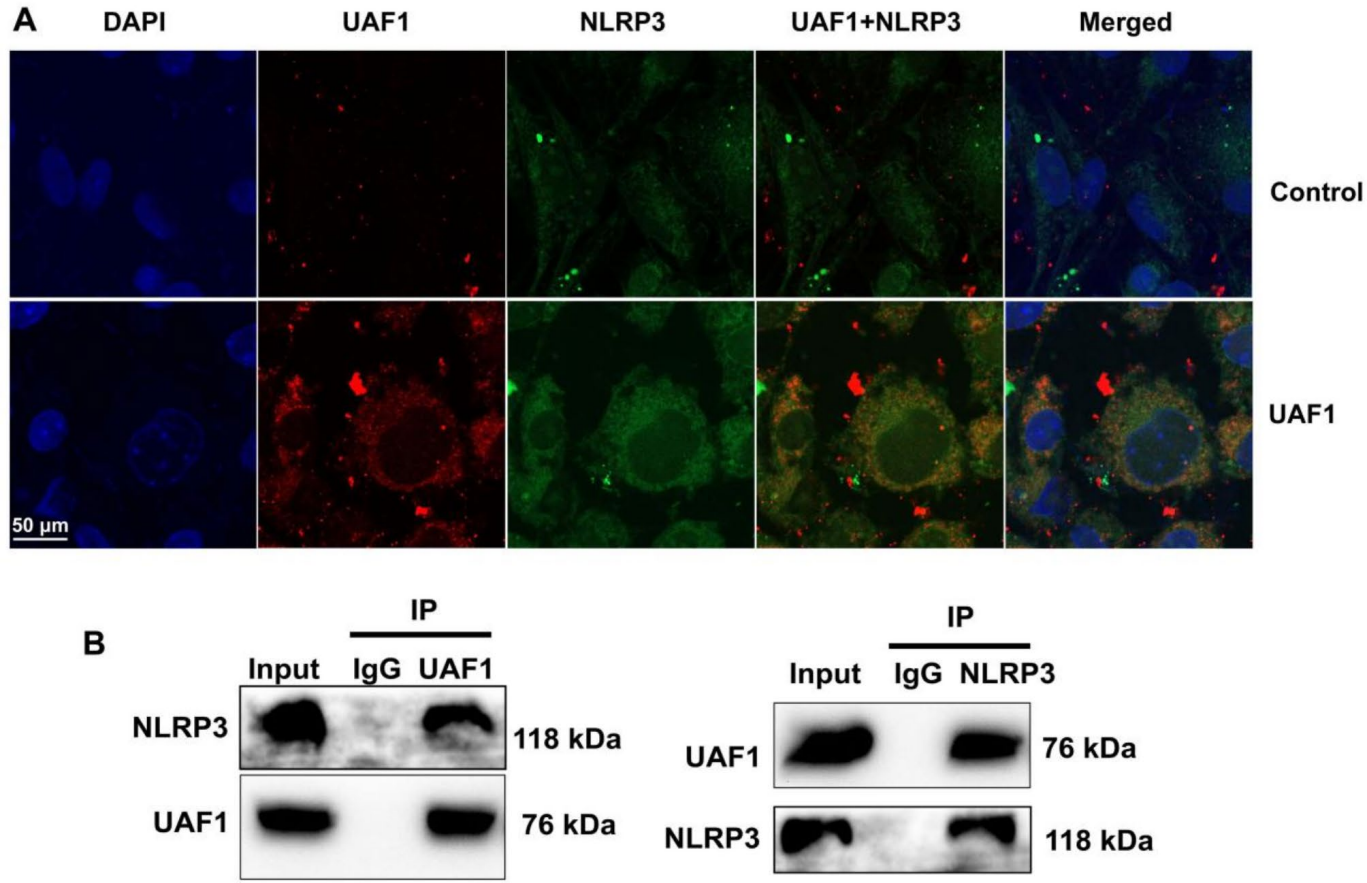
UAF1 interlinking NLRP3 protein expression to reduce NLRP3 ubiquitination by LPS + ATP. UAF1 and NLRP3 expression (immunofluorescence, A), UAF1 interlinking NLRP3 protein (IP, B). Scale bar = 50 μm. **P < 0.05 compared with Control group.

The function of UAF1 in the mouse model of colitis was further examined by NLRP3. The results of immunofluorescence revealed that UAF1 overexpression significantly increased NLRP3 expression level in RAW264.7 macrophages by LPS + ATP (*P* < 0.05, Fig. 7A). The results of ChIP-qPCR indicated that UAF1 was interlinked to NLRP3 protein expression level (*P* < 0.05, Fig. 7B).

### M6A promoted UAF1 stability by METTL3

**Fig. 8:**
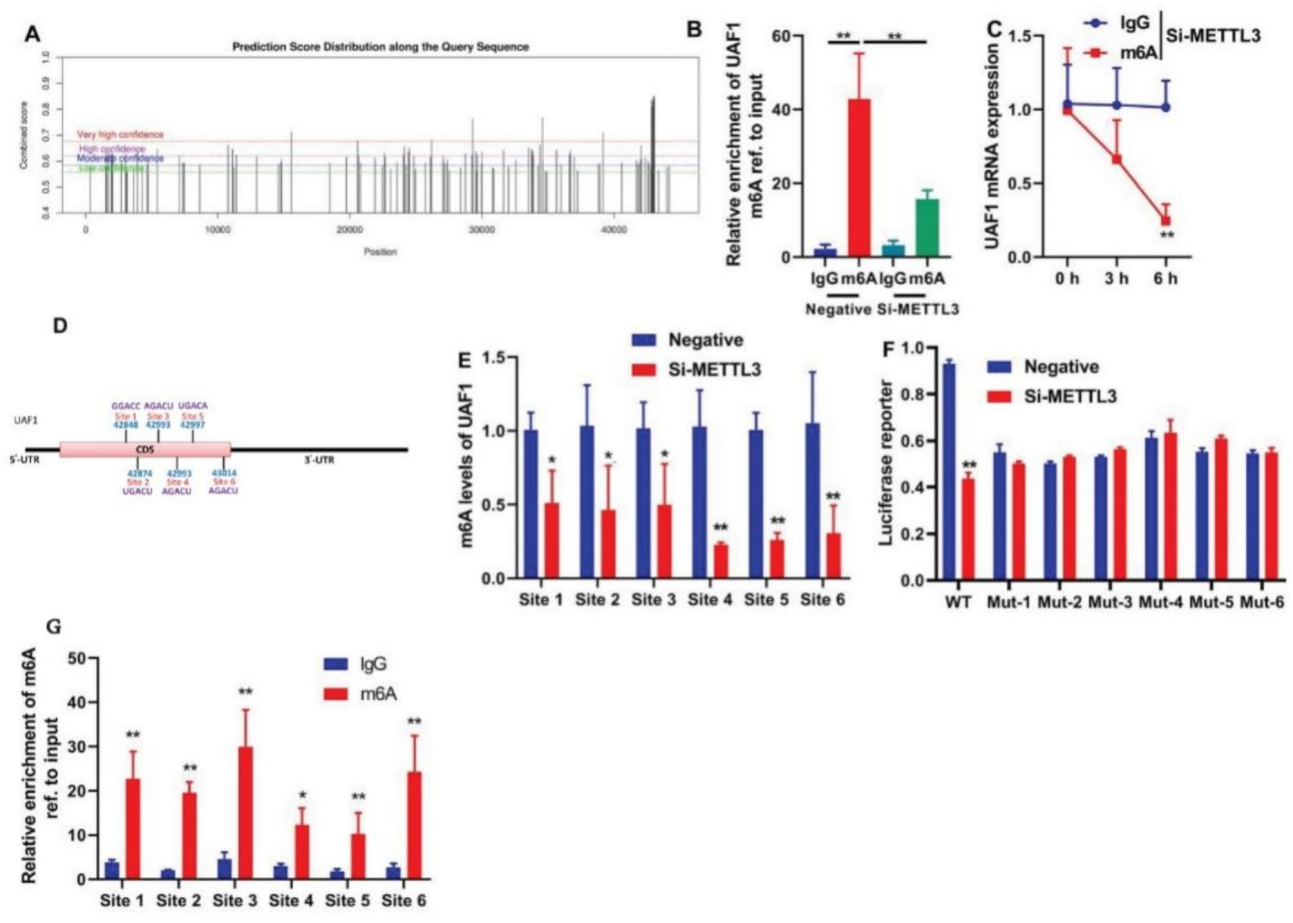
M6A promoted UAF1 stability by METTL3. A prediction score distribution along the query sequence (A), relative enrichment of UAF1 compared between IgG and m6A under negative and SI-METTL3 conditions (B), UAF1 mRNA expression over time for IgG and m6A in SI-METTL3 group (C), six mutation sites in the CDS region (D), mRNA levels of UAF1 at six different sites under negative and SI-METTL3 conditions (E), luciferase activity on wild-type and mutant UAF1 (F), and relative enrichment of UAF1 compared between IgG and m6A at the six sites (G). *P < 0.05 compared with IgG/Negative/METTL3 group; **P < 0.01 compared with IgG/Negative/METTL3 group.

In the study of the methylation mechanism promoting UAF1 stability in colitis, the UAF1 gene was found to possess several potential methylation modification sites. A computational prediction model was applied to generate a distribution of scores along the entire length of the query sequence. The model systematically assessed and scored each position along the sequence, providing a comprehensive depiction of the prediction scores along the queried region (Fig. 8A). RNA immunoprecipitation was employed to selectively isolate UAF1 RNA. The study compared the relative enrichment of UAF1 between IgG and m6A conditions under both negative control and SI-METTL3 experimental setups. RT-qPCR was utilized to precisely quantify the enrichment levels. The relative enrichment of UAF1 compared between IgG and m6A under negative and SI-METTL3 conditions is illustrated in Fig. 8B (*P* < 0.01). A time-course experiment was conducted to monitor the dynamic changes in UAF1 mRNA expression levels. Immunoprecipitation was combined with RT-qPCR to assess UAF1 mRNA expression under SI-METTL3 conditions. Figure 8C depicts UAF1 mRNA expression over time (0, 3, 6 h) for IgG and m6A in SI-METTL3 group (*P* < 0.01). Identification and validation of six mutation sites within the coding sequence (CDS) region of the target gene were achieved, as displayed in Fig. 8D. This involved the use of Sanger sequencing to confirm the presence of mutations at these specific sites. The study concentrated on quantifying mRNA level of the UAF1 gene at six distinct sites. RT-qPCR was applied to assess the mRNA expression at these specified sites under both negative control and SI-METTL3 experimental conditions. The mRNA levels of UAF1 at six different sites under negative and SI-METTL3 conditions are presented in Fig. 8E (*P* < 0.05, *P* < 0.01). Si-METTL3 significantly reduced luciferase activity level of UAF1 in wild-type (WT) (*P* < 0.05, Fig. 8F), while the UAF1 mutant (Mut) did not exhibit any potential methylation modification (*P* < 0.01). The relative enrichment of UAF1 compared between IgG and m6A at the six sites is illustrated in Fig. 8G (*P* < 0.05, *P* < 0.01).

## Discussion

Previous research indicated that the incidence rate of UC in China is 6–11/100,000 person-times per year. The pathogenesis of UC is intricate, with the inflammatory response resulting from an excessive immune response being a noticeable factor in the onset and recurrence of UC.

UC typically presents a recurrent pattern in clinical practice, characterized by prolonged treatment cycles and challenging-to-heal ulcers. This pattern poses a significant risk for the development of colorectal cancer^36^. The conventional treatment strategy for UC is symptom-centered, encompassing clinical drugs, such as biological agents, immunosuppressants, and corticosteroids. However, these treatment methods exhibit limited efficacy and may even lead to side effects^40^. In the present study, an upregulation of UAF1 expression in a mouse model of colitis was found. Nevertheless, the limitation of utilizing only one cell line (RAW264.7 macrophages) is acknowledged. The importance of incorporating additional cell lines to further validate the conclusions is recognized.

UC is characterized by dysregulation of intestinal inflammation^41^. At present, understanding factors, such as the destruction of the intestinal epithelial barrier, the intestinal microenvironment, immune dysfunction, genetics, environmental factors, and oxidative stress is crucial to the onset of UC^42^. As the pace of life quickens and dietary habits become more complex, the incidence of IBD is on the rise. The widespread utilization of colonoscopy technology has significantly improved clinical detection rate. Mesalazine, a 5-aminosalicylic acid drug, is the primary clinical treatment in Western medicine^33,43^. However, challenges persist in clinical practice, including adverse drug reactions, the need for long-term medication, and the risk of relapse after dosage reduction. Additionally, the high cost of biological agents restricts their widespread clinical application^43^. In the classical cell death pathway, cells can undergo pyroptosis mediated by DAMPs, leading to the formation of NLRP3 inflammasome, cleavage of GSDMD protein, and the promotion of cell rupture and death. While activated pyroptosis serves as a self-protective response, excessive pyroptosis can trigger a cascade of inflammatory immune responses, resulting in various diseases. Pyroptosis is associated with colonic inflammation and exacerbation of UC by inducing an excessive inflammatory response. The present study demonstrated that the inhibition of UAF1 suppresses NLRP3-induced IL-1β in the mouse model. UAF1 exhibited to interlink with NLRP3 protein expression level, reducing NLRP3 ubiquitination induced by LPS + ATP.

UC is a disease characterized by chronic inflammation, and abnormal DNA methylation serves as both a consequence and a promoter of this inflammation, particularly in association with severe UC. As the duration of UC extends and abnormal methylation genes increase, there is an evolution towards UC-related colorectal cancer. Detecting and preventing abnormal DNA methylation is crucial for managing UC and preventing cancer development^44^. M6A, a frequent chemical modification in eukaryotic cells, involves the methylation of the sixth N position of adenine in RNA molecules. M6A modification can influence mRNA splicing, nucleation, degradation, stability, and translation processes, thereby regulating gene expression and various biological processes^45,46^. The catalytic residue initiating methylation is the conserved sequence DPPW motif in the METTL3 catalytic site. The large cavity near the intermolecular binding interface of METTL3 may identify RNA substrates, with the SAM binding site situated on one side of its internal cavity^45,46^. This cavity, constituting a highly conserved amino acid sequence in evolution, plays a role in influencing RNA stability, splicing, translation, and nucleation. METTL3 impacts the metabolic activity of various RNA substrates, influencing tumorigenesis, inflammatory reactions, circadian rhythm, angiogenesis, embryonic development, and other processes^45,46^. M6A modification occurs at the post-transcriptional level of RNA and is regulated by methyltransferase, demethylase, and methyl recognition proteins^47,48^. Notably, the present study revealed that the m6A-forming enzyme METTL3 promotes UAF1 stability. It is noteworthy that the present study did not validate these results using clinical samples, and additional models will be employed for validation in the future research.

While the experimental design utilizing a mouse model and RAW264.7 cells provided valuable insights into the role of UAF1 in colitis, it is essential to acknowledge certain limitations that might impact the extrapolation of findings. The study primarily employed a mouse model, and it is crucial to recognize that biological variations between mice and humans may influence the translation of results. Human colitis is a complex condition with distinct features, and caution should be exercised when generalizing the findings of this study to human physiology. RAW264.7 cells were used to assess the molecular mechanisms underlying UAF1’s effects. However, the inherent differences between cell lines and primary cells might limit the direct applicability of our results to human macrophages and other relevant cell types involved in colitis. Colitis encompasses a multifaceted interplay of various cell types, signaling pathways, and the gut microbiome. While the present study concentrated on specific aspects, the holistic nature of colitis at the organism level introduces complexity that may not be fully captured in a model system. Translating findings from preclinical models to clinical applications necessitates careful consideration. Factors, such as dosage, administration routes, and potential side effects of UAF1 inhibitors in human subjects should be thoroughly investigated before expression of clinical relevance. Acknowledging these limitations ensures a more balanced interpretation of the results of this study and underscores the need for further research, potentially involving human samples or clinical trials, to validate the applicability of the findings in a human context.

However, there are some limitations to this study, especially the lack of clinical sample data to support it. The collection of clinical samples is essential to validate the results of laboratory studies. First, clinical samples can directly reflect the real situation in the disease state, helping to confirm whether the phenomenon observed in the laboratory is also present in human patients. While mouse models and in vitro experiments have provided us with initial mechanistic insights, these results need to be validated in human samples to ensure their clinical relevance and reliability. Future work will focus on collecting tissue samples from patients with colitis to detect UAF1 and METTL3 expression levels and their association with inflammatory markers. This will not only help to more fully assess the role of UAF1 and METTL3 in the pathogenesis of colitis, but could also provide key clinical evidence for the development of new treatment strategies. In addition, to better evaluate the clinical significance of UAF1 as a therapeutic target, we plan to compare UAF1 inhibitors with established colitis treatments. Specifically, we will compare the effects of UAF1 inhibitors with known drugs such as mesalazine, tofacitinib, and sulfasalazine in animal models, including improvements in inflammatory markers, pathological changes, and clinical symptoms. This will help to determine the advantages and limitations of UAF1 inhibitors in the treatment of colitis, and provide more theoretical basis and support for future clinical applications.

Given the important role of the gut microbiota in the pathogenesis of colitis, the potential effects of UAF1 inhibition or METTL3 activity on microbial composition and function are also of concern. Imbalances in the gut microbiota, such as dysbiosis, have been shown to be closely associated with the occurrence and development of multiple inflammatory bowel diseases. Therefore, future studies should explore the effects of UAF1 inhibition or METTL3 activity on the gut microbiota, including changes in microbial diversity, increases and decreases in specific flora, and alterations in metabolites. This will contribute to a more complete understanding of the mechanisms of action of UAF1 and METTL3 in colitis.

In conclusion, the present study indicated that the m6A-forming enzyme METTL3 controls UAF1 stabilization, promoting inflammation in the mouse model of colitis by enhancing NLRP3. Furthermore, inhibiting UAF1 emerges as a potential therapeutic strategy for colitis. Further investigations into the specific downstream effectors of the METTL3-UAF1 axis will reveal additional key players in colitis pathogenesis. Exploring the translational potential of UAF1 inhibition in human colitis cases will provide a reliable reference for clinical interventions that harness the therapeutic benefits observed in this mouse model. Beyond colitis, the broader landscape of RNA modifications beckons exploration. Understanding how METTL3-mediated m6A modifications influence other signaling pathways associated with inflammatory diseases will be advantageous for identification of novel therapeutic targets.

## Electronic supplementary material

Below is the link to the electronic supplementary material.

Supplementary Material 1

## Data Availability

The data could be obtained by contacting the corresponding author.

## References

[1] Liu, J. et al. Prdx6-induced inhibition of ferroptosis in epithelial cells contributes to liquiritin-exerted alleviation of colitis. *Food Funct.* (2022). 10.1039/d2fo00945e 35983876

[2] Schoepfer, A. M. et al. Impact of diagnostic delay on disease course in pediatric-versus adult-onset patients with ulcerative colitis: data from the Swiss IBD cohort. *Inflamm. Intest Dis.* **7**, 87–96 (2022). 35979190 10.1159/000520995 PMC9294935

[3] Pu, Z. et al. Systematic understanding of the mechanism and effects of Arctigenin attenuates inflammation in dextran sulfate sodium-induced acute colitis through suppression of NLRP3 inflammasome by SIRT1. *Am. J. Transl. Res.* **11**, 3992–4009 (2019). 31396314 PMC6684881

[4] Sullivan, K. M. et al. Postmarketing colitis cases associated with alpelisib use reported to the US Food and Drug Administration. *JAMA Oncol.* (2022). 10.1001/jamaoncol.2022.3249 PMC9389432 35980660

[5] Li, M. et al. Development of microparticles for oral administration of *Periplaneta americana* extract to treat ulcerative colitis. *Drug Deliv.* **29**, 2723–2733 (2022). 35982644 10.1080/10717544.2022.2112115 PMC9521608

[6] Gu, Q. et al. *Lactiplantibacillus plantarum* ZJ316 fermented milk ameliorates DSS-induced chronic colitis by improving the inflammatory response and regulating intestinal microbiota. *J. Dairy Sci.* (2023). 10.3168/jds.2023-23251 37210370

[7] Lee, Y. et al. *Role of Nox4 in Mitigating Inflammation and Fibrosis in Dextran Sulfate Sodium-induced Colitis* (Cell Mol Gastroenterol Hepatol, 2023). 10.1016/j.jcmgh.2023.05.002 PMC10372905 37207801

[8] He, Z., Liu, J. & Liu, Y. Daphnetin attenuates intestinal inflammation, oxidative stress, and apoptosis in ulcerative colitis via inhibiting REG3A-dependent JAK2/STAT3 signaling pathway. *Environ. Toxicol.* (2023). 10.1002/tox.23837 37209277

[9] Moy, B. M., Shenoy, A. & Aldrich, L. B. A case of nocardiosis in a patient with ulcerative colitis on chronic corticosteroids, infliximab, and upadacitinib. *Clin. Case Rep.* **11**, e7362 (2023). 37207089 10.1002/ccr3.7362 PMC10188892

[10] Dalal, R. S., Sharma, P. P., Bains, K., Pruce, J. C. & Allegretti, J. R. 1-Year comparative effectiveness of Tofacitinib vs Ustekinumab for patients with Ulcerative Colitis and prior antitumor necrosis factor failure. *Inflamm. Bowel Dis.* (2023). 10.1093/ibd/izad087 37209416

[11] Sica, G. S. et al. Surgical management of colon cancer in ulcerative colitis patients with orthotopic liver transplant for primary sclerosing cholangitis. A systematic review. *Eur. J. Surg. Oncol.* (2023). 10.1016/j.ejso.2023.04.021 37210276

[12] Yu, T. et al. Insights into Q-markers and molecular mechanism of Sanguisorba saponins in treating ulcerative colitis based on lipid metabolism regulation. *Phytomedicine* **116**, 154870 (2023). 37207387 10.1016/j.phymed.2023.154870

[13] Sun, S. et al. AMPK activation alleviated DSS-induced colitis by inhibiting ferroptosis. *J. Dig. Dis.* (2023). 10.1111/1751-2980.13176 37210607

[14] Wang, R. et al. CD73 blockade alleviates intestinal inflammatory responses by regulating macrophage differentiation in ulcerative colitis. *Exp. Ther. Med.* **25**, 272 (2023). 37206543 10.3892/etm.2023.11972 PMC10189750

[15] Saito, D. et al. A new endoscopic scoring system corresponding to histological healing using linked color imaging in ulcerative colitis: the SOUL study. *Endosc Int. Open.* **11**, E504–E512 (2023). 37206692 10.1055/a-2067-8943 PMC10191738

[16] Patil, D. T. & Odze, R. D. Biopsy diagnosis of colitis: an algorithmic approach. *Virchows Arch.* **472**, 67–80 (2018). 29177895 10.1007/s00428-017-2274-0

[17] Yuichiro, O. et al. The insoluble excretion of multi-matrix system mesalazine preparations in patients with ulcerative colitis. *BMC Gastroenterol.* **22**, 390 (2022). 35982420 10.1186/s12876-022-02474-9 PMC9389853

[18] Ruiz, P. A. et al. Titanium dioxide nanoparticles exacerbate DSS-induced colitis: role of the NLRP3 inflammasome. *Gut* **66**, 1216–1224 (2017). 26848183 10.1136/gutjnl-2015-310297 PMC5530483

[19] Zhang, W. et al. Network pharmacology for systematic understanding of Schisandrin B reduces the epithelial cells injury of colitis through regulating pyroptosis by AMPK/Nrf2/NLRP3 inflammasome. *Aging (Albany NY)* **13**, 23193–23209 (2021). 34628369 10.18632/aging.203611 PMC8544312

[20] Wu, X. et al. Roseburia intestinalis–derived flagellin ameliorates colitis by targeting miR–223–3p–mediated activation of NLRP3 inflammasome and pyroptosis. *Mol. Med. Rep.* **22**, 2695–2704 (2020). 32700754 10.3892/mmr.2020.11351 PMC7453595

[21] Pu, Z., Shen, C., Zhang, W., Xie, H. & Wang, W. Avenanthramide C from oats protects pyroptosis through dependent ROS-Induced mitochondrial damage by PI3K ubiquitination and phosphorylation in pediatric pneumonia. *J. Agric. Food Chem.* **70**, 2339–2353 (2022). 35119859 10.1021/acs.jafc.1c06223

[22] Qu, S. et al. Akkermansia muciniphila alleviates Dextran Sulfate Sodium (DSS)-induced acute colitis by NLRP3 activation. *Microbiol. Spectr.* **9**, e0073021 (2021). 34612661 10.1128/Spectrum.00730-21 PMC8510245

[23] Liu, P. et al. M(6)A-independent genome-wide METTL3 and METTL14 redistribution drives the senescence-associated secretory phenotype. *Nat. Cell. Biol.* **23**, 355–365 (2021). 33795874 10.1038/s41556-021-00656-3 PMC8035315

[24] Mu, H. et al. METTL3-mediated mRNA N(6)-methyladenosine is required for oocyte and follicle development in mice. *Cell. Death Dis.* **12**, 989 (2021). 34689175 10.1038/s41419-021-04272-9 PMC8542036

[25] Zhang, C. et al. METTL3 and N6-methyladenosine promote homologous recombination-mediated repair of DSBs by modulating DNA-RNA hybrid accumulation. *Mol. Cell.* **79**, 425–442 e427 (2020). 10.1016/j.molcel.2020.06.017 32615088

[26] Chen, L. et al. METTL3-mediated m6A modification stabilizes TERRA and maintains telomere stability. *Nucleic Acids Res.* **50**, 11619–11634 (2022). 36399511 10.1093/nar/gkac1027 PMC9723618

[27] Yu, Z. et al. USP1-UAF1 deubiquitinase complex stabilizes TBK1 and enhances antiviral responses. *J. Exp. Med.* **214**, 3553–3563 (2017). 29138248 10.1084/jem.20170180 PMC5716033

[28] Kim, S. J. et al. ATAD5 suppresses centrosome over-duplication by regulating UAF1 and ID1. *Cell. Cycle* **19**, 1952–1968 (2020). 32594826 10.1080/15384101.2020.1785724 PMC7469630

[29] Liang, Q. et al. A selective USP1-UAF1 inhibitor links deubiquitination to DNA damage responses. *Nat. Chem. Biol.* **10**, 298–304 (2014). 24531842 10.1038/nchembio.1455 PMC4144829

[30] Dexheimer, T. S. et al. Discovery of ML323 as a novel inhibitor of the USP1/UAF1 deubiquitinase complex. In *Probe Reports from the NIH Molecular Libraries Program, Bethesda MD* (2010).

[31] Song, H. et al. UAF1 deubiquitinase complexes facilitate NLRP3 inflammasome activation by promoting NLRP3 expression. *Nat. Commun.* **11**, 6042 (2020). 33247121 10.1038/s41467-020-19939-8 PMC7695691

[32] Liu, Y. et al. Cholecystectomy-induced secondary bile acids accumulation ameliorates colitis through inhibiting monocyte/macrophage recruitment. *Gut Microbes* **14**, 2107387 (2022). 36050867 10.1080/19490976.2022.2107387 PMC9450905

[33] Zhang, W., Wang, W., Xu, M., Xie, H. & Pu, Z. GPR43 regulation of mitochondrial damage to alleviate inflammatory reaction in sepsis. *Aging (Albany NY)* **13**, 22588–22610 (2021). 34584017 10.18632/aging.203572 PMC8507289

[34] Liang, W. et al. FAM3D is essential for colon homeostasis and host defense against inflammation associated carcinogenesis. *Nat. Commun.* **11**, 5912 (2020). 33219235 10.1038/s41467-020-19691-z PMC7679402

[35] Xu, W. et al. Apaf-1 pyroptosome senses mitochondrial permeability transition. *Cell. Metab.* **33**, 424–436 (2021). 33308446 10.1016/j.cmet.2020.11.018

[36] Xiao, F. et al. Bifidobacterium longum CECT 7894 improves the efficacy of Infliximab for DSS-Induced Colitis via regulating the gut microbiota and bile acid metabolism. *Front. Pharmacol.* **13**, 902337 (2022). 35979230 10.3389/fphar.2022.902337 PMC9376241

[37] Jessurun, J. The differential diagnosis of acute colitis: clues to a specific diagnosis. *Surg. Pathol. Clin.* **10**, 863–885 (2017). 29103537 10.1016/j.path.2017.07.008

[38] Yan, Y. et al. A combination of baicalin and berberine hydrochloride ameliorates dextran sulfate sodium-induced colitis by modulating colon gut microbiota. *J. Med. Food* **25**, 853–862 (2022). 35980327 10.1089/jmf.2021.K.0173 PMC9419951

[39] Pu, Z. et al. Using network pharmacology for systematic understanding of geniposide in ameliorating inflammatory responses in colitis through suppression of NLRP3 inflammasome in macrophage by AMPK/Sirt1 dependent signaling. *Am. J. Chin. Med.* **48**, 1693–1713 (2020). 33202149 10.1142/S0192415X20500846

[40] Zhang, H. et al. Corynoline ameliorates dextran sulfate sodium-induced colitis in mice by modulating Nrf2/NF-κB pathway. Immunopharmacol. *Immunotoxicology* **2022**, 1–9 (2022). 10.1080/08923973.2022.2112218 35980837

[41] Jie, F. et al. Kuijieling decoction suppresses NLRP3-Mediated pyroptosis to alleviate inflammation and experimental colitis in vivo and in vitro. *J. Ethnopharmacol.* **264**, 113243 (2021). 32781258 10.1016/j.jep.2020.113243

[42] Lv, Q. et al. Lonicerin targets EZH2 to alleviate ulcerative colitis by autophagy-mediated NLRP3 inflammasome inactivation. *Acta Pharm. Sin B* **11**, 2880–2899 (2021). 34589402 10.1016/j.apsb.2021.03.011 PMC8463273

[43] Mai, C. T. et al. Palmatine attenuated dextran sulfate sodium (DSS)-induced colitis via promoting mitophagy-mediated NLRP3 inflammasome inactivation. *Mol. Immunol.* **105**, 76–85 (2019). 30496979 10.1016/j.molimm.2018.10.015

[44] Du, Y. et al. SUMOylation of the m6A-RNA methyltransferase METTL3 modulates its function. *Nucleic Acids Res.* **46**, 5195–5208 (2018). 29506078 10.1093/nar/gky156 PMC6007514

[45] Wang, C. X. et al. METTL3-mediated m6A modification is required for cerebellar development. *PLoS Biol.* **16**, e2004880 (2018). 29879109 10.1371/journal.pbio.2004880 PMC6021109

[46] Han, J. et al. METTL3 promote tumor proliferation of bladder cancer by accelerating pri-miR221/222 maturation in m6A-dependent manner. *Mol. Cancer* **18**, 110 (2019). 31228940 10.1186/s12943-019-1036-9 PMC6588935

[47] Zhou, D. et al. METTL3/YTHDF2 m6A axis accelerates colorectal carcinogenesis through epigenetically suppressing YPEL5. *Mol. Oncol.* **15**, 2172–2184 (2021). 33411363 10.1002/1878-0261.12898 PMC8333777

[48] Wu, Z. et al. METTL3 counteracts premature aging via m6A-dependent stabilization of MIS12 mRNA. *Nucleic Acids Res.* **48**, 11083–11096 (2020). 33035345 10.1093/nar/gkaa816 PMC7641765

