# Supplementary material for: N6-methyladenosine (m6A)-forming enzyme METTL3 controls UAF1 stability to promote inflammation in a model of colitis by stimulating NLRP3
Source: Sci Rep. 2025 Feb 18;15:5876. doi: 10.1038/s41598-025-88435-0 (PMC11836354; doi:10.1038/s41598-025-88435-0)

Figure 1D Bactin 42 kDa

110 kDa
75 kDa
55 kDa
40 kDa
35 kDa
25 kDa

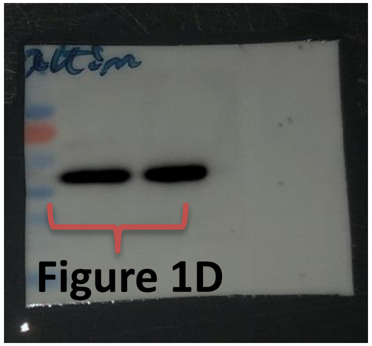

UAF1 76 kDa

110 kDa
75 kDa
55 kDa
40 kDa
35 kDa
25 kDa
20 kDa

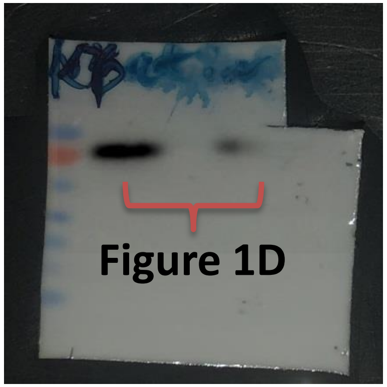

Figure 4D Bactin 42 kDa

110 kDa
75 kDa
55 kDa
40 kDa
35 kDa

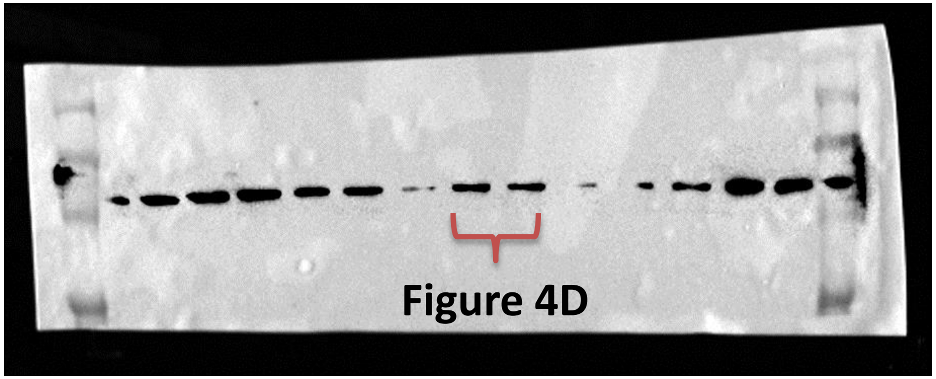

NLRP3 118 kDa

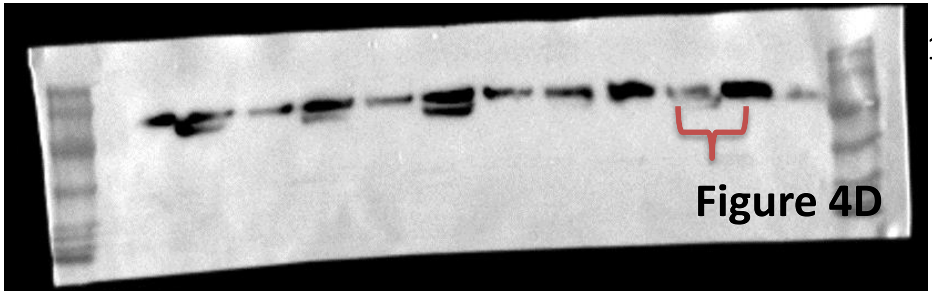

180 kDa
150 kDa
110 kDa
75 kDa
55 kDa

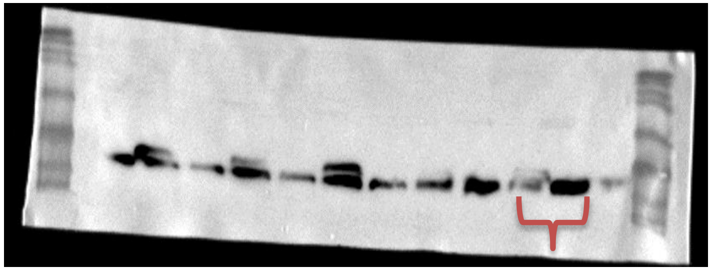

Figure 4D

Figure 5A

UAF1 76 kDa

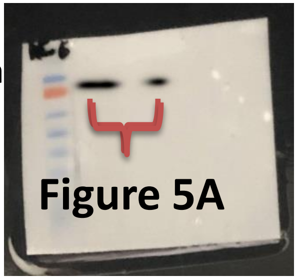

UAF1 76 kDa

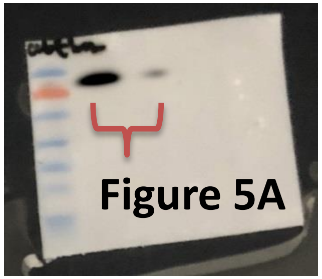

Bactin 42 kDa

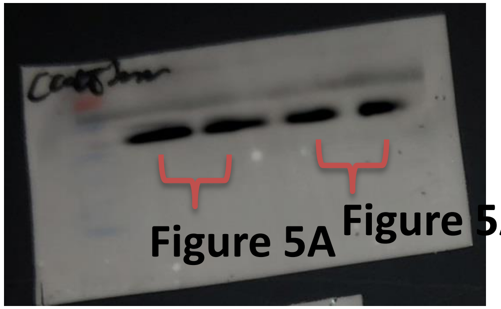

NLRP3 118 kDa

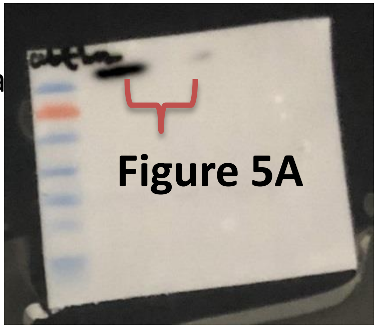

NLRP3 118 kDa

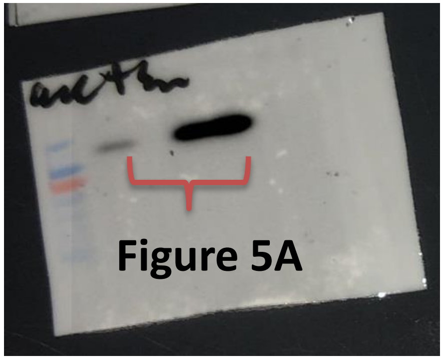

Figure 6A

Bactin 42 kDa

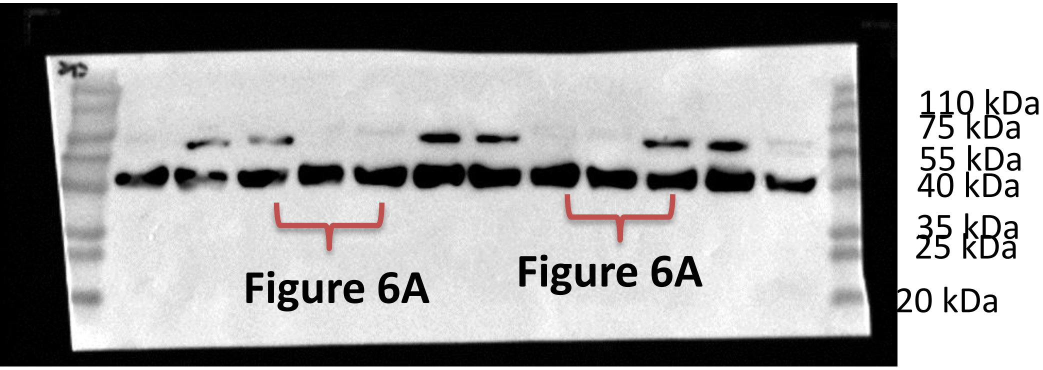

110 kDa
75 kDa
55 kDa
40 kDa
35 kDa
25 kDa
20 kDa

NLRP3 118 kDa

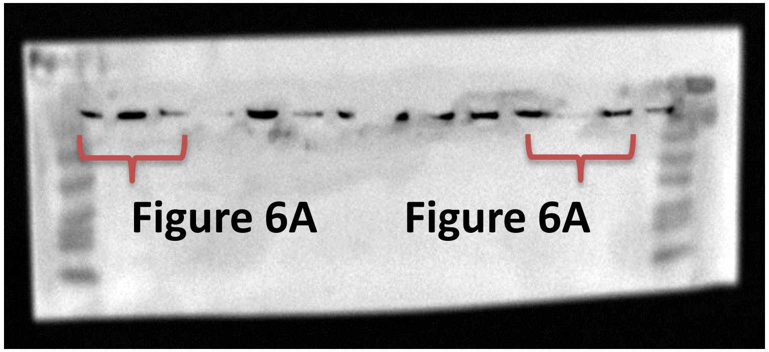

110 kDa
75 kDa
55 kDa
40 kDa
35 kDa
25 kDa
20 kDa

Figure 7B

UAF1 76 kDa

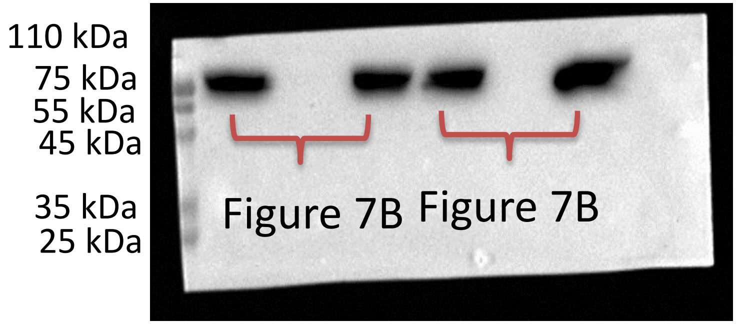

NLRP3 118 kDa

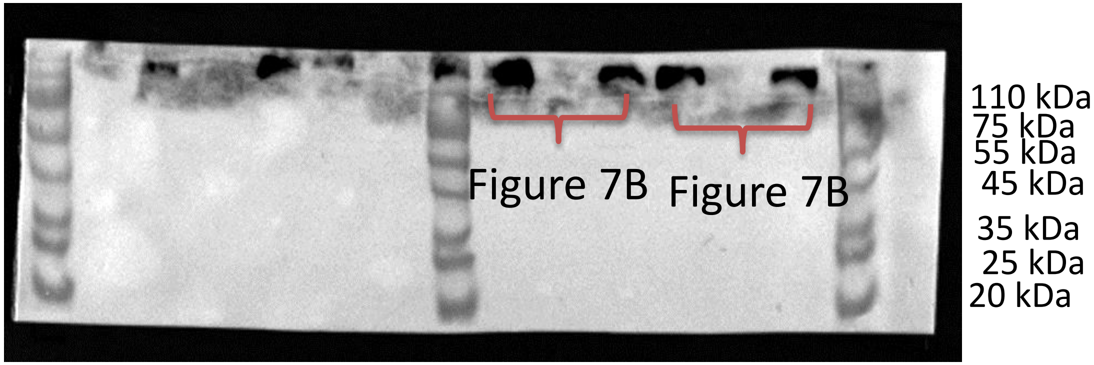

Supplement: Supplementary file 1 — Supplementary Material 1 [file 41598_2025_88435_MOESM1_ESM.pdf]
